# Lipid-sensors, enigmatic-orphan and orphan nuclear receptors as therapeutic targets in breast-cancer

**DOI:** 10.18632/oncotarget.7410

**Published:** 2016-02-15

**Authors:** Enrico Garattini, Marco Bolis, Maurizio Gianni', Gabriela Paroni, Maddalena Fratelli, Mineko Terao

**Affiliations:** ^1^ Laboratory of Molecular Biology, IRCCS-Istituto di Ricerche Farmacologiche “Mario Negri”, Milano, Italy

**Keywords:** nuclear receptors, breast cancer, drug targets, chemo-prevention, treatment

## Abstract

Breast-cancer is heterogeneous and consists of various groups with different biological characteristics. Innovative pharmacological approaches accounting for this heterogeneity are needed. The forty eight human *Nuclear-Hormone-Receptors* are ligand-dependent transcription-factors and are classified into *Endocrine-Receptors, Adopted-Orphan-Receptors* (*Lipid-sensors* and *Enigmatic-Orphans*) and *Orphan-receptors*. Nuclear-Receptors represent ideal targets for the design/synthesis of pharmacological ligands. We provide an overview of the literature available on the expression and potential role played by *Lipid-sensors, Enigmatic-Orphans* and *Orphan-Receptors* in breast-cancer. The data are complemented by an analysis of the expression levels of each selected Nuclear-Receptor in the PAM50 breast-cancer groups, following re-elaboration of the data publicly available. The major aim is to support the idea that some of the Nuclear-Receptors represent largely unexploited therapeutic-targets in breast-cancer treatment/chemo-prevention. On the basis of our analysis, we conclude that the *Lipid-Sensors*, NR1C3, NR1H2 and NR1H3 are likely to be onco-suppressors in breast-cancer. The *Enigmatic-Orphans*, NR1F1 NR2A1 and NR3B3 as well as the *Orphan-Receptors*, NR0B1, NR0B2, NR1D1, NR2F1, NR2F2 and NR4A3 exert a similar action. These Nuclear-Receptors represent candidates for the development of therapeutic strategies aimed at increasing their expression or activating them in tumor cells. The group of Nuclear-Receptors endowed with potential oncogenic properties consists of the *Lipid-Sensors*, NR1C2 and NR1I2, the *Enigmatic-Orphans*, NR1F3, NR3B1 and NR5A2, as well as the *Orphan-Receptors*, NR2E1, NR2E3 and NR6A1. These oncogenic Nuclear-Receptors should be targeted with selective antagonists, reverse-agonists or agents/strategies capable of reducing their expression in breast-cancer cells.

## INTRODUCTION

**Figure 1:**
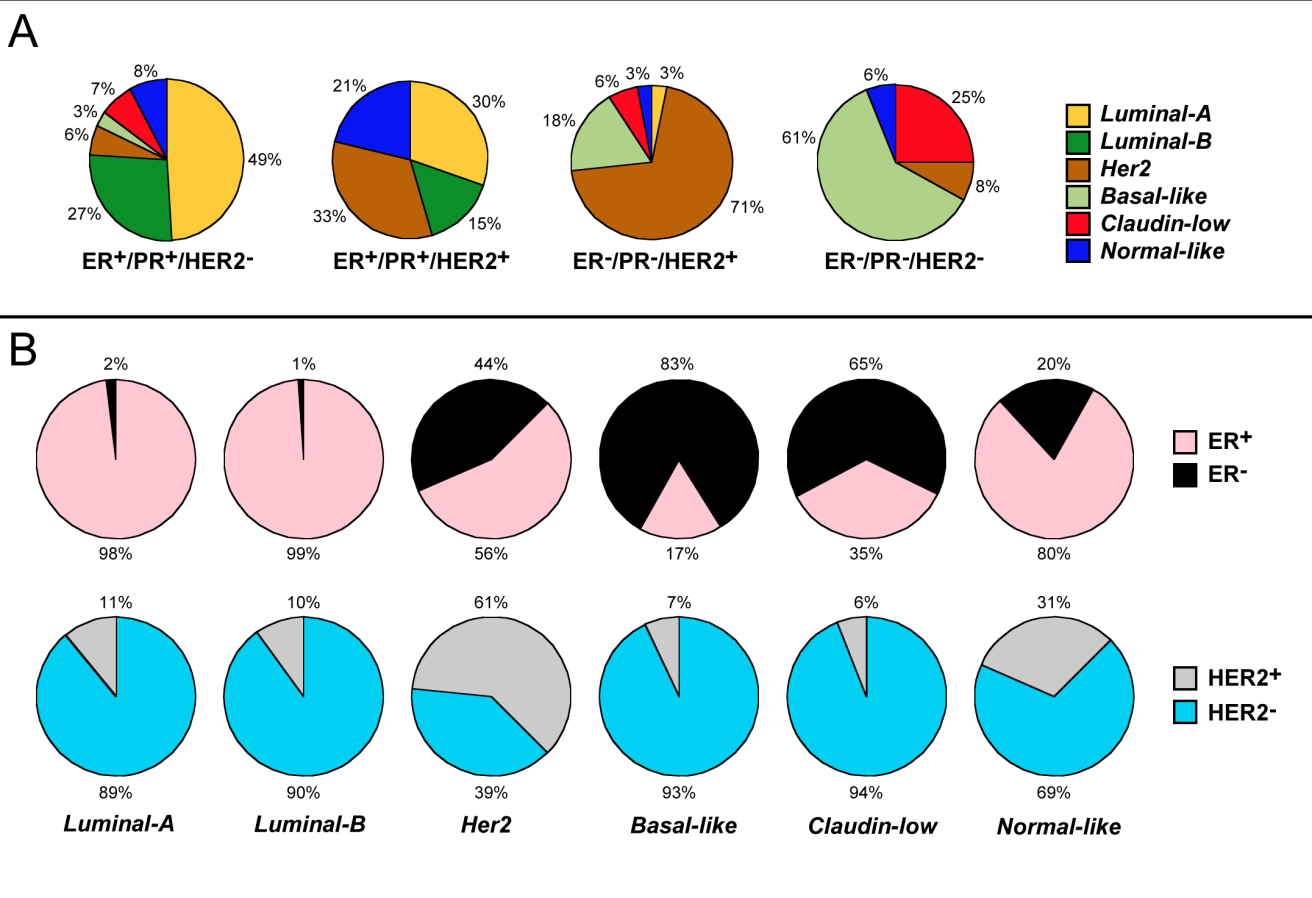
Correspondence between the PAM50 and the traditional classification of breast-cancer **A.** Correspondence between the immuno-histochemical and the molecular/transcriptomic classification of breast cancer. Mammary-tumors are divided in four groups according to the traditional classification based on the immuno-histochemical determination of the ERα, PR and HER2 molecular markers. For each of the four groups, the percentage of cases showing the six indicated transcriptomic phenotypes determined on the basis of a modification of the PAM50 fingerprint is illustrated. **B.** Expression of ERα (Upper circles) and HER2 (Lower circles) among the molecular/transcriptomic subtypes of breast cancer. Mammary tumors are split in six groups according the transcriptomic phenotype as detailed in **A.**. For each of the six groups the percentage of cases showing positivity to ERα is indicated. The results indicates that practically the totality of *Luminal-A* and Luminal-B tumors and the vast majority of the *Normal-like* ones are ER^+^. In contrast, the vast majority of *Basal-like* and *Claudin-Low* tumors, which are aggregated into the PAM50 *Basal* group, are ER^−^. The data were obtained from the following article: Rivenbark AG, O'Connor SM, Coleman WB. Molecular and cellular heterogeneity in breast cancer: challenges for personalized medicine. *Am J Pathol* (2013) 183: 1113-24.

Breast-cancer is heterogeneous and traditionally classified according to the expression of *Estrogen-Receptor-alpha* (NR3A1/ERα), *Progesterone-Receptor* (NR3C3/PR) and/or HER2, the *ERBB2* gene product. With these markers, breast-cancer is subdivided into ER-positive (*ER^+^*), HER2-positive (*HER2^+^*) and triple-negative tumors. Genomic/transcriptomic data indicate that the number of breast-cancer groups is larger than originally assumed. The PAM50 gene-expression fingerprint classifies mammary-tumors in five groups, *Basal, Her2, Luminal-A, Luminal-B* and *Normal-like*, each having different biological characteristics and drug-sensitivity [1]. A more recent classification further split PAM50 *Basal* cancer into *Basal-like* and *Claudin-low* tumors (Figure 1).

**Figure 2:**
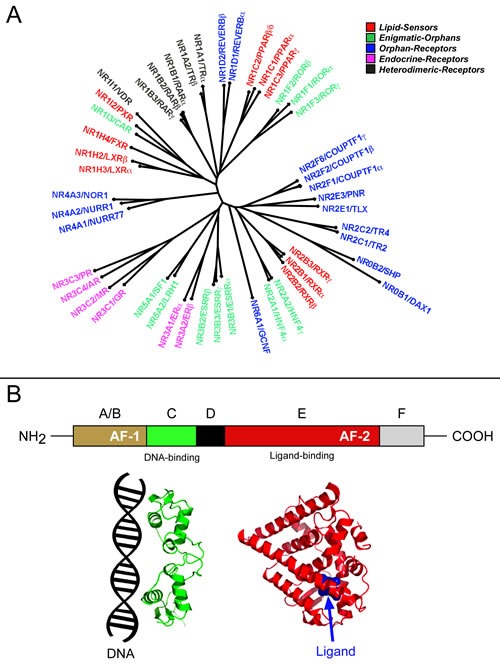
Phylogenetic tree and schematic structure of the human nuclear steroid receptors **A.** The panel illustrates an unrooted phylogenetic tree of the human nuclear steroid receptors. The phylogenetic tree was obtained following alignment of the amino acid sequences of the indicated nuclear receptors using the Clustal-omega algorithm (Sievers F, Wilm A, Dineen DG, Gibson TJ, Karplus K, Li W, Lopez R, McWilliam H, Remmert M, Söding J, Thompson JD, Higgins D. Fast, scalable generation of high-quality protein multiple sequence alignments using Clustal Omega Molecular Systems Biology 7 Article number: 539 doi:10.1038/msb.2011.75) **B.** The schematic structure of a typical nuclear receptor (NR) is shown in the upper diagram. The N-terminal A- and B-regions are responsible for the ligand-independent activation function (AF-1). The C-region contains the DNA-binding domain, while the unstructured D-region connects the C- and E-regions. The E-region containing the ligand-binding pocket is responsible for the ligand-dependent activation function (AF-2). The function of the C-terminal F-region is undefined. The tridimensional structure of the C- and E-regions contained in a representative NR (ERα) is illustrated in the lower part of the figure.

The superfamily of human *Nuclear-Hormone-Receptors* (NRs) consists of forty eight members, which are ligand-dependent transcription-factors controlling the expression of specific gene-sets [2]. However, this is unlikely to represent their sole function, as transcription-independent activities are known for various NRs. NRs are encoded by distinct genes which, in many cases, give rise to different splicing-/protein-variants (Tables 1, 2, 3). The amino-acid sequences of human NRs are highly similar (Figure 2A) and contain up to six conserved structural regions (Figure 2B). The N-terminal A- and B-regions are responsible for the ligand-independent transcriptional-activation-function (AF-1). The C-region contains the DNA-binding domain, while the D-region is unstructured. The E-region contains the ligand-binding-domain and is responsible for the ligand-dependent transcriptional-activation-function (AF-2). The function of the C-terminal ill-conserved F-region is undefined. As the C- and E-regions are particularly important and structurally conserved, their position in NRs is indicated in Tables 1, 2, 3.

**Table 1:** Human lipid sensors

*Lipid-Sensors*								
Gene	Chr (No)	Exons (No)	Protein-variant	Prot (aa)	DBD (aa No)	LBD (aa No)	Ligands	Predicted action in breast-cancer
*NR1C1* (PPARα) *Peroxysome-Proliferator-Activated-Receptor-α*	22	8	NP_001001928	468	101-184	201-467	1-Palmitoyl-2-oleoyl-sn-glycero-3-phosphocholine (PUBCHEM:65167)(end. agonist) Chakravarthy MV *et al*, *Cell*(2009)138:476-88 CP775146 (PUBCHEM:10410059)(synth. agonist) Kane CD *et al*, *Mol Pharmacol* (2009)75:296-306 GW6471 (PUBCHEM:446738)(synth. antagonist) Muller MQ *et al*, *J Med Chem* (2009)52:2875-9	Mixed results
*NR1C2* (PPARβ/δ) *Peroxysome-Proliferator-Activated-Receptor-β/δ*	6	8 7 6 8	NP_006229 NP_001165290 NP_001165291 NP_803184	441 402 343 361	73-156 34-117 41-58 73-156	173-440 134-401 75-342 173-359	PGI2 (PUBCHEM:23702145)(end. agonist) Gupta RA *et al*, *Proc Natl Acad Sci U S A* (2000)97:13275-80 15-HETE (PUBCHEM:9966861)(end. agonist) Naruhn S *et al*, *Mol Pharmacol* (2010) 77:171-84 Fatty-acids (end. agonists) Barish GD *et al*, *J Clin Invest* (2006)116:590-7 GW0742 (PUBCHEM:9934458)(synth. agonist) Sznaidman L *et al*, *Bioorg Med Chem Lett* (2003)13:1517-21 GSK3787 (PUBCHEM:2800647)(**synth. antagonist**) Palkar PS *et al*, *Mol Pharmacol* (2010)78:419-30	Oncogenic action *Her2* tumors
*NR1C3* (PPARγ) *Peroxysome-Proliferator-Activated-Receptor-γ*	3	8 7	NP_619725 NP_056953	477 505	110-193 138-221	209-476 237-504	15-deoxy-Δ12,14-PGJ2 (PUBCHEM:5311211)(end. agonist) Kotta-Loizou IC *et al*, *Anticancer Agents Med Chem* (2012)12:1025-44 Unsatur. fatty-acids (end. agonists) **Troglitazone (PUBCHEM:5591)(synth. agonists) Kodera Y *et al*, *J Biol Chem*** (2000)275:33201-4 BADGE (PUBCHEM:3479589)(synth. antagonist) Bishop-Bailey D *et al*, *Br J Pharmacol* (2000) 131:651-4	**Onco-suppressive action**
*NR1H2* (LXRβ) *Liver-X-Receptor-β*	19	10 9	NP_009052 NP_001243576	461 364	67-163 -	224-459 127-362	27-hydroxycholesterol (PUBCHEM:123976)(end. agonist) Song C *et al*, *Steroids* (2000)65:423-7 Oxysterols (end. agonists) Zhao C and Dahlman-Wright K, *J Endocrinol* (2010)204:233-40 **GW3965 (PUBCHEM:447905)(synth. agonist) Collins JL *et al*, J Med Chem(2002)45:1963-6** GSK2033 (PUBCHEM:46203250)(synth. antagonist) Zuercher WJ *et al*, *J Med Chem* (2010)53:3412-6	**Onco-suppressive action**
*NR1H3* (LXRα) *Liver-X-Receptor-α*	11	10 10 9 9	NP_001238863 NP_005684 NP_001123574 NP_001123573	453 447 402 387	84-184 78-178 33-133 78-187	216-451 210-445 165-400 210-385	27-hydroxycholesterol (PUBCHEM:123976)(end. agonist) Song C *et al*, *Steroids* (2000)65:423-7 Oxysterols (end. agonists) Zhao C and Dahlman-Wright K, *J Endocrinol* (2010)204:233-40 **GW3965 (PUBCHEM:447905)(synth. agonists) Collins JL *et al*, J Med** Chem(2002)45:1963-6 GSK2033 (PUBCHEM:46203250)(synth. antagonist) Zuercher WJ *et al*, *J Med Chem* (2010)53:3412-6	**Onco-suppressive action**
*NR1H4* (FXR) *Farnesoid-X-Receptor*	12	11 11 10 9 9	NP_005114 NP_001193908 NP_001193907 NP_001193921 NP_001193922	472 476 425 482 486	124-207 124-211 127-149 134-217 134-221	247-467 251-471 200-420 252-470 256-474	Deoxycholate (PUBCHEM:23668196)(end. agonist) Silva J *et al*, *J Lipid Res* (2006) 47:724-33 Chenodeoxycholic-acid (PUBCHEM:10133)(end. agonist) Makishima M *et al*, *Science* (1999) 284:1362-5 GW4064 (PUBCHEM:9893571)(synth. agonist) Maloney PR *et al*, *J Med Chem* (2000) 43:2971-4 guggulsterone (PUBCHEM:6439929)(synth. antagonist) Owsley E and Chiang JY, *Biochem Biophys Res Commun* (2003) 304:191-5	Mixed results
*NR1I2* (PXR) *Pregnane-X-Receptor*	3	9 9 9	NP_003880 NP_148934 NP_071285	434 397 473	40-127 40-127 79-166	143-428 143-391 182-467	Rifampicin (PUBCHEM:6913622)(synth. agonist) Moore LB *et al*, *J Biol Chem* (2000) 275:15122-7 SR12813 (PUBCHEM:446313) (synth. agonist) Lemaire G *et al*, *Mol Pharmacol* (2007) 72:572-81 Meclizine (PUBCHEM:4034)(synth. antagonist) Lau AJ *et al*, *J Pharmacol Exp* Ther(2011) 336:816-26	Oncogenic action

The table contains basic information on the characteristics of the *Lipid-Sensors* group of nuclear receptors (NRs). The first column lists the human NRs considered in the review article. The official symbol of each NR is indicated in italics, while the original alias of each protein product is indicated in parenthesis. The full name of each NR is indicated underneath in italics. The second column from the left lists the human chromosome (Chr) each NR maps to. The number of exons encoding the transcripts giving rise to the corresponding NR protein-variant is indicated in the third column. The fourth column lists the accession number of each NR protein-variant. The amino acid (aa) length of each NR protein variant, the position of the DNA-binding domain (DBD) and the ligand-binding domain (LBD) are indicated in columns five, six and seven, respectively. Column eight contains a list of representative endogenous (end.) and synthetic (synth.) agonists, antagonists and reverse agonists for each NR along with an appropriate reference. The chemical structures of the listed molecules can be found in the PUBCHEM database with the use of the PUBCHEM-CID accession numbers provided. The PUBCHEM chemical structure is not available in the case of the NR2E1 agonists Ccrp-1, -2 and -3. When possible, the predicted onco-suppressive (bold) or oncogenic (black-boxed) action of the corresponding NR is indicated in the last column on the right. Synthetic agonists and antagonists of potential therapeutic interested targeting onco-suppressive and oncogenic NRs, respectively, are marked in bold and boxed in black. Finally, in the few cases where supportive data are available, the type of breast-cancer which is predicted to represent a preferential target of the NR is listed in the last column.

**Table 2:** Human enigmatic orphans

*Enigmatic-Orphans*								
*NR1F1* (RORα) *RAR-Related-Orphan-Receptor-α*	15	11 12 11	NP_599023 NP_599022 NP_002934	523 556 548	66-160 99-193 91-185	272-523 305-544 297-536	ATRA (PUBCHEM: 444795)(end.agonist) Stehlin-Gaon C *et al*, *Nat Struct Biol* (2003)10: 820-5. Melatonin (PUBCHEM:896)(end.agonist) Dai J *et al*, *Mol Cell Endocrinol* (2001)176:111-20 **SR1078 (PUBCHEM:17980288)(synth. agonist) Kojetin D *et al*, *ACS Chem Biol* (2011) 6:131-4** SR1001 (PUBCHEM:44241473)(synth.antagonist) Solt LA *et al*, *Nature*(2011)472: 491-4	**Onco-suppressive action** ER^−^ tumors
*NR1F2* (RORβ) *RAR-Related-Orphan-Receptor-β*	9	10	NP_008845	459	3-97	210-450	Unknown end. agonists N-[5-(2-chloro-benzoyl)-4-(3-chlorophenyl)-thiazol-2-yl]-2-(4-ethanesulfonyl-phenyl)-acetamide (PUBCHEM:8813095)(synth. reverse agonist) Gege C *et al Bioorg Med Chem Lett* (2014)24:5265-7	Unknown
*NR1F3* (RORγ) *RAR-Related-Orphan-Receptor-γ*	1	10 11	NP_001001523 NP_005051	497 518	4-97 24-118	247-485 268-506	7β,27-dihydroxycholesterol (PUBCHEM:24895774)(end.agonist) Soroosh P *et al*, *Proc Natl Acad Sci U S A* (2014)111:12163-8. SR2211 (PUBCHEM:51035449)(synth. antagonist) Kumar N *et al*, *ACS Chem Biol* (2012) 7:672-7 N-[5-(2-chloro-benzoyl)-4-(3-chlorophenyl)-thiazol-2-yl]-2-(4-ethanesulfonyl-phenyl)-acetamide (PUBCHEM:8813095)(synth. reverse agonist) Gege C *et al Bioorg Med Chem Lett* (2014)24:5265-7	Oncogenic Action ER^−^ tumors
*NR1I3* (CAR) *Constitutive-Androstane-Receptor*	1	9 9 9 9 8 8 8 8 8 8 8 7 7	NP_005113 NP_001070950 NP_001070948 NP_001070937 NP_001070940 NP_001070949 NP_001070947 NP_001070939 NP_001070942 NP_001070944 NP_001070945 NP_001070938 NP_001070943	348 357 352 340 324 314 319 309 296 311 306 280 267	11-82 11-82 11-82 11-82 7-53 11-82 7-53 11-82 11-82 7-53 7-53 7-53 7-53	106-346 106-355 106-350 106-311 77-322 106-312 77-317 106-307 106-267 77-282 77-277 77-278 77-238	Androstenol (PUBCHEM:101989)(end. antagonist) Makinen J *et al*, *Biochem J* (2003) 376:465-72 CITCO (PUBCHEM:9600409)(synth. agonist) Maglich JM *et al*, *J Biol Chem* (2003) 278:17277-83. TCPOBOP (PUBCHEM:5382)(synth. agonist) Yamamoto Y et al, *PLoS One* (2010) 5:e10121 BDBM50422490 (CHEMBL141998) (synth. antagonist) Jyrkkarinne J *et al*, *J Med Chem* (2003) 46: 4687-95.	Unknown
*NR2A1* (HNF4α) *Hepatocyte-Nuclear-Factor-4α*	20	10 11 10 8 10 10 8	NP_001245284 NP_001274112 NP_849180 NP_849181 NP_787110 NP_001025174 NP_001274113	467 449 464 417 452 442 395	53-128 35-110 60-135 60-135 38-113 38-113 35-110	144-366 126-348 151-373 151-373 129-351 129-351 126-348	**Linoleic-acid (PUBCHEM:5280450)(end. agonist) Yuan X *et al*, *PLoS One* (2009) 4:e5609.**	**Onco-suppressive action**
*NR2A2* (HNF4γ) *Hepatocyte-Nuclear-Factor-4γ*	8	10	NP_004124	445	49-124	140-361	Fatty-acids (end. agonists) Sladek F, *Mol Cell* (2002) 10:219-21	Unknown
*NR3B1* (ESRRα) *Estrogen-Related-Receptor-α*	11	7	NP_001269379	423	73-168	197-420	Diethylstilbestrol (PUBCHEM:448537)(end. agonist) Ariazi EA and Jordan VC, *Curr Top Med Chem* (2006) 6:203-15 SR16388 (PUBCHEM:54612678)(synth. antagonist) Duellman SJ *et al*, *Biochem Pharmacol* (2010) 80:819-26 XCT790 (PUBCHEM:6918788)(synth. antagonist) Lanvin O *et al*, *J Biol Chem* (2007) 282:28328-34	Oncogenic Action *Her2* tumors *Basal* tumors
*NR3B2* (ESRRβ) *Estrogen-Related-Receptor-β*	14	10	NP_004443	508	98-193	212-431	Diethylstilbestrol (PUBCHEM:448537)(end. agonist) Ariazi EA and Jordan VC, *Curr Top Med Chem* (2006) 6:203-15 **DY131 (PUBCHEM:5497124)(synth. agonist) Yu DD and Forman BM, *Bioorg Med Chem Lett* (2005) 15:1311-3**	**Onco-suppressive action**
*NR3B3* (ESRRγ) *Estrogen-Related-Receptor-γ*	1	9 8 7 8	NP_001230448 NP_001230447 NP_001429 NP_001230436	435 470 458 396	99-195 127-223 122-218 99-156	214-433 249-468 237-456 175-394	Diethylstilbestrol (PUBCHEM:448537)(end. agonist) Ariazi EA and Jordan VC, *Curr Top Med Chem* (2006) 6:203-15 **GSK4716 (PUBCHEM:5331325)(synth. agonist) Zuercher WJ *et al*, *J Med Chem* (2005) 48: 3107-9** **GSK9089 (PUBCHEM:5497124)(synth. agonist) Zuercher WJ *et al*, *J Med Chem* (2005) 48: 3107-9**	**Onco-suppressive action**
*NR5A1* (SF-1) *Steroidogenic-Factor-1*	9	7	NP_004950	461	13-105	223-459	phosphatidic-acid (PUBCHEM: 5283523)(end. agonist) Krylova IN *et al*, *Cell* (2005) 120:343-55 phosphatidyl-choline (PUBCHEM: 5287971)(end. agonist) Sablin EP *et al*, *Mol Endocrinol* (2009) 23: 25-34 GSK8470 (PUBCHEM:10883540)(synth. agonist) Whitby RJ *et al*, *J Med Chem* (2006) 49: 6652-5 AC-45594 (PUBCHEM:25641)(synth. antagonist) Del Tredici AL *et al*, *Mol Pharmacol* (2008) 73: 900-8	Unknown
*NR5A2* (LRH-1) *Liver-Receptor-Homolog-1*	1	8 7 7	NP_995582 NP_001263393 NP_003813	541 469 495	86-178 14-106 40-132	342-390 229-469 255-495	phosphatidic-acid (PUBCHEM: 5283523)(end. agonist) Krylova IN *et al*, *Cell* (2005) 120:343-55 GSK8470 (PUBCHEM:10883540)(synth. agonist) Whitby RJ *et al*, *J Med Chem* (2006) 49: 6652-5	Oncogenic action

The table contains basic information on the *Enigmatic-Orphans* group of nuclear receptors (NRs). The first column lists the human NRs considered in the review article. The official symbol of each NR is indicated in italics, while the original alias of each protein product is indicated in parenthesis. The full name of each NR is indicated underneath in italics. The second column from the left lists the human chromosome (Chr) each NR maps to. The number of exons encoding the transcripts giving rise to the corresponding NR protein-variant is indicated in the third column. The fourth column lists the accession number of each NR protein-variant. The amino acid (aa) length of each NR protein variant, the position of the DNA-binding domain (DBD) and the ligand-binding domain (LBD) are indicated in columns five, six and seven, respectively. Column eight contains a list of representative endogenous (end.) and synthetic (synth.) agonists, antagonists and reverse agonists for each NR along with an appropriate reference. The chemical structures of the listed molecules can be found in the PUBCHEM database with the use of the PUBCHEM-CID accession numbers provided. The structure of the NR1I3 antagonist, BDBM50422490, is available in the CHEMBL database as indicated. When possible, the predicted onco-suppressive (bold) or oncogenic (black-boxed) action of the corresponding NR is indicated in the last column on the right. Synthetic agonists and antagonists of potential therapeutic interested targeting onco-suppressive and oncogenic NRs, respectively, are marked in bold and boxed in black. Finally, in the few cases where supportive data are available, the type of breast-cancer which is predicted to represent a preferential target of the NR is listed in the last column.

**Table 3:** Human orphan receptors

*Orphan-Receptors*								
*NR0B1* (DAX-1) *Dosage-sensitive-sex reversal- Adrenal-hypoplasia-critical-region-on-chromosome-X-gene-1*	X	2	NP_000466	470	-	231-464	Unknown	**Onco-suppressive action**
*NR0B2* (SHP) *Small-Heterodimeric-Partner*	1	2	NP_068804	257	-	32-254	**CD437 (PUBCHEM:135411)(synth.agonist) Farhana L et al, *Cancer Res* (2007) 67:318-25**	**Onco-suppressive action**
*NR1D1* (REV-ERBα) *Related-to-vERBα*	17	8	NP_068370	614	127-215	418-611	Heme (PUBCHEM:444097)(end.agonist) Raghuram S *et al*, *Nat Struct Mol Biol* (2007) 14:1207-13 GSK4112 (PUBCHEM:50905018) (synth.agonist) Meng QJ *et al*, *J Cell Science* (2008) 121:3629-35 SR9009 (PUBCHEM:57394020) (synth.agonist) Solt LA *et al*, *Nature* (2012) 485:62-8	**Onco-suppressive action**
*NR1D2* (REV-ERBβ) *Related-to-vERBβ*	3	8	NP_005117	579	98-186	389-577	**Heme (PUBCHEM:444097)(end.agonist) Raghuram S *et al*, *Nat Struct Mol Biol* (2007) 14:1207-13** **SR9009 (PUBCHEM:57394020)(synth.agonist) Solt LA *et al*, *Nature* (2012) 485:62-8** SR8278 (PUBCHEM:53393127)(synth.antagonist) Kojetin D *et al*, *ACS Chem Biol* (2011) 6:131-4	Unknown
*NR2C1* (TR2) *Testicular-Receptor-2*	12	14 12 12	NP_003288 NP_001120834 NP_001027458	603 483 467	108-194 108-194 108-194	368-589 368-465 368-464	Unknown	Unknown
*NR2C2* (TR4) *Testicular-Receptor-4*	3	14 15	NP_001278623 NP_003289	596 615	112-198 131-217	361-582 380-601	Unknown	Unknown
*NR2E1* (TLX) *Drosophila-Tailless-Homolog*	6	9 9	NP_003260 NP_001273031	385 422	8-99 45136	187-354 191-406	Ccrp-1, -2 and -3 (synth. agonists) Benod C *et al*, *PLoS One* (2014)9:e99440	Oncogenic Action
*NR2E3* (PNR) *Photo-specific-Nuclear-Receptor*	15	7 8	NP_057430 NP_055064	367 410	39-130 39-130	192-367 192-397	Unknown	Oncogenic Action
*NR2F1* (COUPTF1α) *COUP-Transcription-Factor-1α*	5	3	NP_005645	423	86-158	184-419	Unknown	**Onco-suppressive action**
*NR2F2* (COUPTF1β) *COUP-Transcription-Factor-1β*	15	3 3 3	NP_066285 NP_001138628 NP_001138627	414 261 281	79-151 - -	183-414 24-258 44-278	Unknown Pyridaben (PUBCHEM:91754)(**synth. antagonist**) *bindingDB database* (http://www.bindingdb.org)	**Onco-suppressive action**
*NR2F6* (COUPTF-1γ) *COUP-Transcription-Factor-1γ*	19	4	NP_005225	404	56-128	165-400	Unknown	Oncogenic action
*NR4A1* (NURR77) *Nuclear-Receptor-Related-77*	12	8 8 8	NP_001189163 NP_001189162 NP_002126	652 611 598	319-393 278-352 264-339	415-652 374-611 409-459	Unknown	**Onco-suppressive action**
*NR4A2* (NURR1) *Nuclear-Receptor-Related-1*	2	8	NP_006177	598	260-335	409-459	Unknown	Unknown
*NR4A3* (NOR1) *Nuclear-Orphan-Receptor-1*	9	7 8 5	NP_775292 NP_008912 NP_775291	637 626 443	301-375 290-364 290-364	407-637 396-626 -	**PGA2 (PUBCHEM:5280880)(end. agonist) Kagaya S *et al*, *Biol Pharm Bull* (2005) 28:1603-7** **6-mercaptopurine (PUBCHEM:667490)(synth. agonist) Wansa KD *et al*, *J Biol Chem* (2003) 278:24776-90**	**Onco-suppressive action**
*NR6A1* (GCNF) *Germ-Cell-Nuclear-Factor*	9	10 10	NP_201591 NP_001480	480 475	52-141 48-137	256-467 251-462	Unknown	Oncogenic Action

The table contains basic information on the characteristics of the *Orphan-Receptors* group of nuclear receptors (NRs). The first column lists the human NRs considered in the review article. The official symbol of each NR is indicated in italics, while the original alias of each protein product is indicated in parenthesis. The full name of each NR is indicated underneath in italics. The second column from the left lists the human chromosome (Chr) each NR maps to. The number of exons encoding the transcripts giving rise to the corresponding NR protein-variant is indicated in the third column. The fourth column lists the accession number of each NR protein-variant. The amino acid (aa) length of each NR protein variant, the position of the DNA-binding domain (DBD) and the ligand-binding domain (LBD) are indicated in columns five, six and seven, respectively. Column eight contains a list of representative endogenous (end.) and synthetic (synth.) agonists, antagonists and reverse agonists for each NR along with an appropriate reference. The chemical structures of the listed molecules can be found in the PUBCHEM database with the use of the PUBCHEM-CID accession numbers provided. The PBCHEM chemical structure is not available in the case of the NR2E1 agonists Ccrp-1, -2 and -3. When possible, the predicted onco-suppressive (bold) or oncogenic (black-boxed) action of the corresponding NR is indicated in the last column on the right. Synthetic agonists and antagonists of potential therapeutic interested targeting onco-suppressive and oncogenic NRs, respectively, are marked in bold and boxed in black. Finally, in the few cases where supportive data are available, the type of breast-cancer which is predicted to represent a preferential target of the NR is listed in the last column.

Human *NRs* are classified into *Endocrine-Receptors, Adopted-Orphan-Receptors* and *Orphan-receptors* according to the presence/absence of identified endogenous-ligands [3] (Tables 1, 2, 3). *Endocrine-Receptors* are divided into *Steroid-Receptors* and *Heterodimeric-Receptors*. *Steroid-Receptors* include *Estrogen-Receptor-α* (NR3A1/ERα) and *Estrogen-Receptor-β* (NR3A2/ERβ), *Progesterone-Receptor* (NR3C3/PR), *Androgen-Receptor* (NR3C4/AR), *Mineralcorticoid-Receptor* (NR3C2/MR) and *Glucocorticod-Receptor* (NR3C1/GR). The *Vitamin-D-Receptor* (NR1I1/VDR), *Thyroid-Receptor-alpha* (NR1A1/TRα), *Thyroid-Receptor-beta* (NR1A2/TRβ), *Retinoic-Acid-Receptor-alpha* (NR1B1/RARα), *Retinoic-Acid-Receptor-beta* (NR1B2/RARβ) and *Retinoic-Acid-Receptor-gamma* (NR1B3/RARγ) are *Heterodimeric-Receptors*. *Adopted-Orphan-Receptors* are split into *Lipid-sensors* and *Enigmatic-Orphans*.

Innovative pharmacological approaches taking into account the heterogeneity of breast cancer are needed. NRs represent ideal targets for the design/synthesis of pharmacological ligands. Indeed, the E-region accommodates only organic compounds and the design of new agonists/antagonists is facilitated by crystallographic data and functional screening assays. This article provides an overview of the literature available on the expression and role played by *Lipid-sensors, Enigmatic-Orphans* and *Orphan-Receptors* in breast-cancer induction/progression. *Steroid-Receptors, Heterodimeric-Receptors* and the *Lipid-sensors, Retinoid-X-Receptors* (NR2B1/RXRα, NR2B2/RXRβ, NR2B3/RXRγ) are excluded from our analysis given the wealth of data on their therapeutic significance in breast-cancer [4, 5]. The data are complemented by an analysis of the expression levels of each NR in the PAM50 breast-cancer groups, following re-elaboration of the data in the TCGA site (The-Cancer-Genome-Atlas: http://cancergenome.nih.gov). The review supports the idea that some of the *NRs* considered represent unexploited therapeutic-targets in breast-cancer treatment/chemo-prevention.

## LIPID-SENSORS

The active transcriptional forms of *Lipid-sensors* are *RXR* heterodimers*. NR1C1-C3, NR1H2-4* and *NR1I2* are permissive heterodimeric partners, as the simultaneous presence of *NR1* and *RXR* ligands results in cooperative responses[6].

### NR1C1 (PPARα:peroxysome-proliferator -activated-receptor-α), NRC2 (PPARβ/δ:peroxysome-proliferator-activated-receptor-β/δ) and NRC3 (PPARγ:peroxysome-proliferator-activated-eeceptor-)

**Figure 3:**
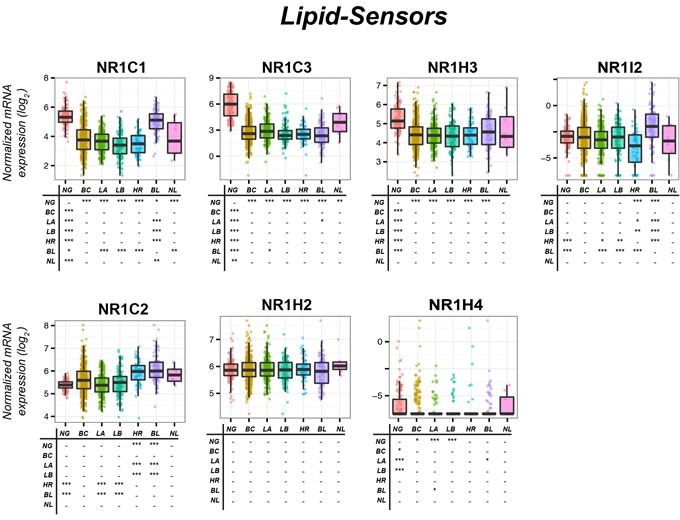
Expression of Lipid-sensors in normal mammary glands and breast-cancer tissue The box-plots illustrate the expression of the indicated mRNAs belonging to the *Lipid-sensors* family of nuclear receptors (NRs) in normal mammary-glands (*NG*), all breast-cancers (*BC*) and *Luminal-A* (*LA*), *Luminal-B* (*LB*), *HER2* (*HR*), *Basal* (*BL*) and *Normal-like* (*NL*) PAM50 mammary-tumors. Underneath each box-plot, the tables show significant differences in the mRNA expression levels of each NR between the indicated groups. The results were obtained from the data available in the TCGA (The Cancer Genome Atlas; http://cancergenome.nih.gov). Normalization, quantification and statistical analysis on raw sequencing counts was performed using the Limma/Voom (http://bioconductor.org) package in R statistical environment. * (adjusted *p* < 0.01), ** (adjusted *p* < 0.001), *** (adjusted *p* < 0.0001).

*NR1C1*, NRC2 and NR1C3 control lipid homeostasis. NR1C3 is the most studied member of the NR1C subfamily, given its relevance in obesity/diabetes. The two NR1C2 shortest protein-variants present with incomplete DNA- and Ligand-binding domains, respectively (Table 1). The only identified endogenous NR1C1 activator is 1-palmitoyl-2-oleoyl-sn-glycerol-3-phosphocholine (POGP). Prostaglandin-PGI2, 15-hydroxyeicosatetraenoic-acid (15-HETE) and polyunsaturated fatty-acids are recognized endogenous NRIC2 ligands. Certain prostaglandins and fatty-acids act as endogenous NR1C3 ligands. Some of the numerous synthetic agonists/antagonists available are listed in Table 1. NR1C1-mRNA (UNIGENE-Hs.103110), NR1C2-mRNA (UNIGENE-Hs.696032) and NR1C3-mRNA (UNIGENE-Hs.162646) expression is ubiquitous and the transcripts are detectable in mammary-glands, albeit at very low levels in the case of NR1C1. Relative to the normal counterpart, NR1C1 and NR1C3 mRNAs are down-regulated in all PAM50-classified breast-cancers (Figure 3). *Basal* and *Normal-like* tumors show the highest NR1C1 and NR1C3 levels, respectively. In contrast, mammary-tumors express higher NR1C2 mRNA levels than the normal counterpart, due to up-regulation in *Her2, Basal* and *Normal-like* cancers.

It is unclear whether NR1C1 has oncogenic or an oncosuppressive properties, as mixed evidence is available. Ligand-dependent NR1C1 activation increases the proliferation of ER^−^/*MDA-MB231* and ER^+^/*MCF-7* cells [7]. In addition, the NRIC1-ligand, Wy14643, promotes tumor-mammosphere formation[8]. Despite this evidence, the NR1C1-agonist, hydroxyeicosatetraenoic-acid, induces apoptosis in ER^+^/ and ER^−^/breast-cancer cells and this effect is potentiated by RXR-selective ligands [9]. NR1C1 onco-suppressive action may involve breast-cancer stromal components. Indeed, NR1C1-activation in mammary-tumor cells suppresses *Hypoxia-Inducible-Factor-1-α signaling*, reduces *Vascular-Endothelial-Growth-Factor* secretion and tube formation by endothelial cells, suggesting anti-angiogenic effects [10]. NR1C1 anti-tumor activity may be of particular relevance in ER^+^/breast-cancer, since anti-estrogens induce NR1C1-mRNA in ER^+^/*MCF-7* cells and ERα over-expression into ER^−^/*MDA-MB-231* cells decreases NR1C1-mRNA [11].

In ER^+^/*MCF-7* and ER^+^/*T47D* breast-cancer cells, NR1C2-activation by GW501516 stimulates proliferation and angiogenic responses [10]. In addition, the NR1C2-antagonist, SR13904, inhibits cell-growth and -survival [12]. Two NR1C2-inverse-agonists (ST247 = methyl 3-(N-(4-(hexylamino)-2-methoxyphenyl)sulfamoyl)thiophene-2-carboxylate and DG172 = (Z)-2-(2-bromophenyl)-3-(4-(4-methylpiperazin-1-yl)phenyl)acrylonitrile dihydrochloride) inhibit serum- and TGFβ-induced invasion of ER^−^/*MDA-MB231* cells into three-dimensional matrixes, suggesting that NR1C2 favors tumor-cell dissemination [13]. A role in ST247/DG172 action is played by *Angiopoietin-Like-4* down-regulation. NR1C2 oncogenic action is supported by data obtained in MMTV-PPARβ/δ transgenic-models of mammary carcinogenesis where the NR1C2-agonist, GW501516, reduces tumor latency [14]. Further support comes from transgenic-mice over-expressing *3-Phosphoinositide-Dependent-Kinase-1*, a protein regulated by NR1C2, where GW501516 accelerates tumorigenesis [15]. NR1C2 oncogenic action may involve the EGFR/ERBB2 pathway *via* FABP5 (Fatty-Acid-Binding-Protein-5), which delivers ligands to NR1C2. In ER^+^/*MCF-7* cells, EGFR/ERBB2-dependent proliferative responses are accompanied by FABP5 induction [16]. In MMTV-ErbB2/HER2 mice, spontaneously developing breast-cancer, FABP5 ablation relieves activation of EGFR downstream signals, down-regulates NR1C2 target-genes involved in cell-proliferation and suppresses tumor-growth [17]. The retinoid pathway may also be involved in NR1C2 oncogenic activity, as NR1C2 is bound/activated by ATRA [18]. Indeed, ATRA delivery to NR1C2 and RARs is dependent on FABP5 and CRABP-II, respectively. In ATRA-resistant MMTV-neu transgenic-model of breast-cancer, decreasing FABP5/CRABP-II ratio diverts ATRA from NR1C2 to RAR and suppresses tumor-growth [19]. Although all this is consistent with an oncogenic action of NR1C2 in breast-cancer, there is also evidence to the contrary. The NR1C2-agonist, GW501516, inhibits *MCF-7* cells growth [20] and NR1C2 over-expression/activation reduces clonogenicity and *in-vivo* growth of ER^+^/*MCF-7* and ER^−^/*MDA-MB231* cells [21]. In conclusion, the majority of the available data indicates that NR1C2 is pro-oncogenic in breast-carcinoma [22].

NR1C3 levels are associated with improved clinical outcome and represent a prognostic factor for overall-survival in ER^+^/breast-cancer patients, suggesting onco-suppressive properties [23]. The synthetic NR1C3-agonists, thiazolidinediones, suppress mammary-tumor growth *in-vitro* and *in-vivo* [24–26]. Thiazolidinedione-activated NR1C3 interferes with ERα, *Signal-Transducer-and-Activator-of-Transcription-5B* and *Nucler-Factor-kappaB*, inhibiting the proliferation of ER^+^/ and ER^−^/cells which undergo differentiation [27] and apoptosis [28]. In addition, thiazolidinediones inhibit TGFβ signaling, which suppresses breast-cancer early-development [25]. Finally, NR1C3-agonists reduce mammary-tumor angiogenesis and invasion [29]. In rats, the NR1C3-ligand, GW7845, inhibits carcinogen-induced breast-cancer [26]. Troglitazone prevents 7,12-dimethylbenz(a)anthracene-induced transformation of murine breast-tissue [30] and *NR1C3* heterozygous-deletion causes greater susceptibility to mammary-tumor development after exposure to the same anthracene-related compound [31]. The action of NR1C3 stimulation is not limited to breast-cancer prevention and extends to treatment [32]. The only exception to the *in-vivo* data supporting NR1C3 onco-suppressive properties is represented by a study showing that the NR acts as a tumor-promoter in a transgenic-model of breast carcinogenesis *via* interference with the WNT-pathway [33]. A small-sized clinical-trial reports that patients with metastatic breast-cancer fail to show any benefit from troglitazone administration [34]. An equally small and recent trial demonstrates that administration of rosiglitazone between the time of diagnostic biopsy and definitive surgery is well-tolerated although it does not alter breast-cancer cell-proliferation [35].

### NR1H2 (LXRβ:liver-X-receptor-β) and NR1H3 (LXRα:liver-X-receptor-α)

NR1H2 and NR1H3 are involved in the metabolism/homeostasis of cholesterol/lipids/bile-acids/steroid-hormones and modulate innate immunity [36]. NR1H2 and NR1H3 are activated by oxysterols, like 27-hydroxy-cholesterol (Table 1). There is cross-talk between NR1H2/NR1H3 and NR1H4/NR1F1, as NR1H4 activation down-regulates NR1H3, while NR1H3 and NR1F1 antagonize each other in terms of transcriptional activity [36, 37]. NR1H2 (UNIGENE-Hs.432976) and NR1H3 (UNIGENE-Hs.438863) mRNA expression is ubiquitous. The largest amounts of NR1H3 mRNA are observed in adipose-tissue. According to the TCGA dataset, normal mammary-glands contain high NR1H2 and NR1H3 mRNA levels (Figure 3). No difference in NR1H2 expression is observed among the PAM50 groups of breast-cancer and normal tissue. By contrast, NR1H3 is down-regulated in all PAM50 tumor groups relative to the normal mammary-gland.

As for the potential role of NR1H2 and NR1H3 in breast-cancer, little and contrasting evidence is available. NR1H2/NR1H3 ligands secreted by tumor cells inhibit CCR7 expression on maturing dendritic cells, impairing immune-surveillance and favoring tumor growth [38]. In mouse breast-cancer models, 27-hydroxycholesterol augments ER-dependent mammary-tumor growth and increases NR1H2/NR1H3-dependent metastasis [39]. The two effects require conversion of cholesterol into 27-hydroxycholesterol by CYP27A1 [40]. The data obtained in these mouse models contrast with the observations made in some breast-cancer cells, where NR1H2/NR1H3 activation reduces proliferation with down-regulation of genes involved in cell cycle progression, DNA replication and other cell-growth-related processes [41, 42]. In ER^+^/breast-tumors the NR1H2/NR1H3 growth-inhibitory action may result from systemic effects, as the two NRs control hepatic estrogen biosynthesis. In the liver, NR1H2/NR1H3 stimulation results in sulfotransferase (an enzyme critical for estrogen deactivation) induction which inhibits breast-cancer growth in xenografted mice [43]. Non-cell autonomous growth-inhibitory effects are also suggested by *in-vitro* results, as culture medium from NR1H2/NR1H3 activated macrophages causes growth-inhibition and apoptosis of breast-cancer cells [44].

In conclusion, NR1H2/NR1H3 activation may represent a strategy for the treatment/chemoprevention of breast-cancer, although caution should be exercised, since NR1H2/NR1H3-agonists may favor the metastatic spread of tumor cells [39].

### NR1H4 (FXR:farnesoid-X-receptor)

NR1H4 transcriptional activity is stimulated by bile-acids, such as chenodeoxycholic acid [45, 46]. Adrenal-glands, kidney, liver and intestine express the highest NR1H4-mRNA levels (UNIGENE-Hs.282735), although the transcript is present also in mammary-glands. With the exception of the *Normal-like* group, all PAM50 mammary-tumor sub-types express smaller NR1H4 amounts than the normal counterpart (Figure 3). In breast-cancer, NR1H4-protein levels are associated with ER-status and luminal markers [47].

Data on the role played by NR1H4 in mammary-tumors are contrasting. As bile-acids are a risk factor for post-menopausal breast-cancer [48], their high concentrations in breast-cysts/plasma of mammary-tumor patients [49] suggest a role for NR1H4 in disease induction/progression. Consistent with this hypothesis, the NR1H4 agonist, deoxycholate, promotes survival and favors migration of ER^−^/*MDA-MB-231* cells, while the inverse-agonist, guggulsterone, exerts opposite effects [50]. Similarly, farnesol, another NR1H4-agonist, exerts mitogenic effects in ER^+^/*MCF-7* cells, but not in ER^−^/*MDA-MB231* cells [47]. Mitogenesis may require binding/activation of ERα by NR1H4 [47]. NR1H4-dependent proliferation of ER^+^/breast-cancer cells is stimulated by estrogen deprivation, which recapitulates menopause and aromatase-inhibitor treatment [47]. In spite of the evidence indicating that NR1H4 is oncogenic, particularly in ER^+^/breast-cancer, there is also evidence supporting the idea that NR1H4-activation is anti-oncogenic. High concentrations of the GW4064 agonist induce apoptosis of ER^+^/*MCF-7* and ER^−^/*MDA-MB468* cells [51]. Chenodeoxycholic-acid and GW4064 inhibit growth of tamoxifen-resistant ER^+^/cells. Interestingly, chenodeoxycholic-acid reduces EGF-induced growth of these cells *via* inhibition of the HER2 pathway [52]. On the basis of these data, it is currently difficult to establish whether NR1H4 is endowed with oncogenic or onco-suppressive properties in breast cancer and whether targeted therapeutic strategies should be aimed at inhibiting or activating the receptor.

### NR1I2 (PXR:pregnane-X-receptor)

NR1I2 plays a role in xenobiotic metabolism and is activated by various synthetic ligands (Table 1) [53]. NR1I2 regulates the expression of genes involved in xenobiotic metabolism and transport [54]. NR1I2-mRNA highest levels are observed in liver although considerable amounts are measurable also in mammary-glands (UNIGENE-Hs.7303). Relative to normal tissue, NR1I2-mRNA is down-regulated in *Her2* tumors, while the opposite is true in *Basal* tumors (Figure 3). The TCGA data on the expression of the NR1I2 transcript in the PAM50 breast cancer subtypes are only partially in line with the reported inverse relationship between NR1I2-mRNA expression and ER-positivity [55]. NR1I2 represents a negative prognostic marker in breast-cancer, as NR1I2-protein levels correlate with labeling-index, histologic-grade and lymph-node-status [56]. In addition, NR1I2-protein over-expression and nuclear localization is associated with infiltrative-carcinoma recurrence [56]. NR1I2 plays a role in breast-cancer cell resistance to anti-tumor agents. In ER^+^/*MCF-7* cells, NR1I2 is involved in induced resistance to tamoxifene *via* up-regulation of *Multidrug-Resistance-Associated-protein-2*, a membrane-transporter controlling drug efflux [57]. In ER^+^/*MCF-7* and *ER^−^/MDA-MB231* cells, SR12813 causes docetaxel resistance and induction of the drug-resistance genes, *Multidrug-Resistance-1* and *Breast-cancer-Resistance-Protein B* [58]. Thus, NR1I2-inhibition should be considered in the treatment of tumors with acquired resistance to anti-hormones and chemotherapy.

## ENIGMATIC-ORPHANS

The family of *Enigmatic-orphans* contains *NRs* whose endogenous ligands are ill-defined or unknown and consists of eleven members.

### NR1F1 (RORα:RAR-related-orphan-receptor-α), NR1F2 (RORβ:RAR-related-orphan-receptor-β) and NR1F3 (ROR:RAR-related-orphan-receptor-γ)

**Figure 4:**
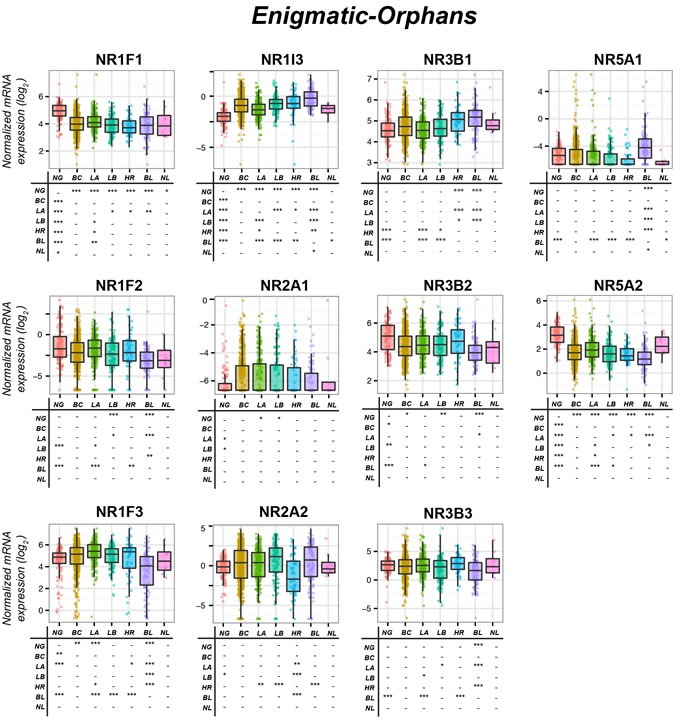
Expression of Enigmatic-Orphan receptors in normal mammary glands and breast-cancer tissue The box-plots illustrate the expression of the indicated mRNAs belonging to the *Enigmatic-Orphans* family of nuclear receptors (NRs) in normal mammary-glands (*NG*), all breast-cancers (*BC*) and the *Luminal-A* (*LA*), *Luminal-B* (*LB*), *HER2* (*HR*), *Basal* (*BL*) and *Normal-like* (*NL*) PAM50 mammary-tumors. Underneath each box-plot, the tables show significant differences in the mRNA expression levels of each NR between the indicated groups. The results were obtained from the data available in the TCGA (The Cancer Genome Atlas; http://cancergenome.nih.gov). Normalization, quantification and statistical analysis on raw sequencing counts was performed using the Limma/Voom (http://bioconductor.org) package in R statistical environment. * (adjusted *p* < 0.01), ** (adjusted *p* < 0.001), *** (adjusted *p* < 0.0001).

NR1F1, NR1F2 and NR1F3 control circadian rhythms [59]. NR1F2 plays also a role in lineage specification of CD4^+^ T-helper into Th17 cells [60]. NR1F1, NR1F2 and NRF3 bind to the regulatory regions of target-genes (ROR-Response-Elements) as monomers [61]. ATRA and melatonin are endogenous NR1F1-agonists, while 7β,27-dihydroxycholesterol is an endogenous NR1F3-ligand (Table 3). Only synthetic agonists/antagonists targeting NR1F1 and NR1F3 are available. The highest NR1F1-mRNA levels are observed in skin, muscle and adrenal-glands (UNIGENE-Hs.560343), while NR1F2-mRNA expression is restricted to eyes and adrenal-glands (UNIGENE-Hs.494178) and NR1F3-mRNA is ubiquitous (UNIGENE-Hs.256022). NR1F1 and NR1F3 are expressed in the mammary-gland, while NRF2 is under-represented in the organ. All PAM50-classified breast-cancer types express lower NR1F1-mRNA levels than the normal tissue (Figure 4). *Luminal-A* and *Normal-like* tumors presents with the largest NR1F1 amounts. Similarly, NR1F2 is down-regulated in most cancer subgroups relative to the normal tissue*. Basal* tumors are the poorest NR1F2 source. Unlike NR1F1 and NR1F2, larger NR1F3-mRNA amounts are present in mammary-tumors than in normal glands.

NR1F1 is a growth stimulator in ER^+^/cells, while it is an inhibitor in ER^−^/cells [62]. NR1F1 may stimulate ER^+^/cell-growth *via* ERα binding/activation or aromatase induction [63]. Besides inhibiting growth, NR1F1 exerts anti-invasive actions in ER^−^/cells and tumors [64–66]. In ER^−^/cells and xenografts, transcriptional *Semaphorin-3F* induction *via* a *ROR-Response-Element* contributes to NR1F1 anti-invasive and growth-inhibitory activities [64]. In addition, NR1F1 binds *E2F1-transcription-factor*, inhibits *E2F1*-acetylation and transcriptional activity, reducing ductal epithelial-cell proliferation [66]. No studies on NR1F2 in breast-cancer are available and the number of publications involving NR1F3 is limited [64, 67, 68]. High NR1F3 expression is associated with an increase in metastasis-free survival [67]. It is also possible that NR1F3 expression enhances mammary-tumors aggressiveness reducing T-lymphocytes immune-surveillance [64]. In conclusion, NRF1 induction/activation and NRF13 suppression/inhibition may be of therapeutic use, particularly in ER^−^/breast-cancer

### NR1I3 (CAR:constitutive-androstane-receptor)

NR1I3 is characterized by a large number of protein variants (Table 2). NR1I3 high basal activity is inhibited by steroids related to androstenol [69] and is involved in drug-metabolism and energy-homeostasis [70]. NR1I3 and NR1I2 control an overlapping set of genes and their physiological function seems to be redundant, although NR1I3 plays a unique role in bilirubin clearance [71]. NR1I3 is activated by 4-bis[2-(3,5-dichloropyridyloxy)]benzene (TCPOBOP) directly or indirectly by phenobarbital *via* cytoplasm-to-nucleus translocation [72]. The only known synthetic NR1I3-agonist is (4-chlorophenyl)imidazo[2,1-b][1,3]thiazole-5-carbaldehyde-O-(3,4-dichlorobenzyl)oxime (CITCO), while various synthetic NR1I3-inhibitors are available. NR1I3-mRNA expression is limited to liver and kidney (UNIGENE-Hs.349642) and very low NR1I3-mRNA levels are detectable in the mammary-gland. NR1I3 is up-regulated in all mammary-tumor sub-types, with the sole exception of *Normal-like* cancer (Figure 4). There are no studies on NR1I3 in breast cancer indicating whether it could be oncogenic or not.

### NR2A1(HNF4α:hepatocyte-nuclear-factor-4α) and NR2A2 (HNF4γ:hepatocyte-nuclear-factor-4)

NR2A1 binds as a homodimer to target genes [73] and its transcriptional activity may be regulated by endogenous ligands such as linoleic acid [74]. NR2A2 interacts with the same DNA target-sites recognized by NR2A1 [75] and it is bound/activated by endogenous fatty-acids, which are difficult to replace with synthetic molecules [76]. NR2A1-mRNA expression is restricted to liver, kidney, small intestine and stomach (UNIGENE-Hs.116462), while NR2A2-mRNA is present in kidney, stomach and muscle (UNIGENE-Hs.241529). Very small NR2A1 and NR2A2 mRNA amounts are detectable in mammary-glands. NR2A1 levels are slightly higher in *Luminal-A* and *Luminal-B* cancers relative to normal tissue (Figure 4). *Her2* tumors contain the smallest NR2A2 amounts. In mammary-glands, NR2A1 has been the object of few studies [77–81], while no studies on NR2A2 are available. NR2A1 may have oncosuppressive properties [77], as it is down-regulated in mammary epithelial-cells undergoing epithelial-to-mesenchymal transition (EMT).

### NR3B1 (ERRα:estrogen-related-receptor-α), NR3B2 (ERRβ: estrogen-related-receptor-β) and NR3B3 (ERRγ:estrogen-related-receptor-γ)

NR3B1, NR1B2 and NRB3 show structural similarity with ERs [82]. Diethylstilbestrol is a non-selective endogenous NR3B-agonist (Table 2). NR3Bs bind to ERRE (Estrogen-Related-Response-Elements) in target-genes. Although it was originally thought that NR3Bs and ERα control common target-genes, a recent study demonstrates that NR3B1 and ERα share only a minor fraction of targets [83]. NR3B1-mRNA (UNIGENE-Hs.110849) and NRB3-mRNA (UNIGENE-Hs.444225) are ubiquitously expressed, while NRB2-mRNA expression is restricted (eye and muscle; UNIGENE-Hs.435845). NR3B1 regulates circadian-clocks, influencing metabolic homeostasis and locomotor-activity [84–86].

In mammary-glands, NR3B1 and NR3B2 are highly expressed, while NR3B3 levels are negligible (Figure 4). Normal tissues and all PAM50-classified breast-cancers express similar amounts of NR3B2-mRNA and NR3B3-mRNA. By converse, *HER2-like* and *Basal-like* mammary-tumors contain larger NR3B1-mRNA amounts than the normal tissue, which is consistent with a positive correlation between NR3B1 and HER2 expression [87]. In line with this, HER2/MAPK/AKT activation causes NR3B1 phosphorylation/activation in HER2^+^/*BT474* cells [88]. In addition, NR3B1 activates transcription of the genes contained in the *ERBB2* amplicon observed in the majority of HER2^+^ breast tumors, possibly explaining the delay in tumor development observed following *NR3b1* knock-out in a mouse model of ERBB2-initiated mammary cancerogenesis [89]. NR3B1 is a negative prognostic factor for breast-tumors, being associated with increased recurrence-risk and adverse clinical-outcome [90]. Consistent with NR3B1 oncogenic action, NR3B1-antagonists reduce the size of ER^+^/ and ER^−^/xenografts [91], while NR3B1 knock-down diminishes *in-vitro* migration and *in-vivo* growth of ER^−^/*MDA-MB-231* cells [91]. The pathways underlying NR3B1 pro-oncogenic action are obscure and may vary in ER^+^/ and HER2^+^/tumors. *WNT* inhibitors suppress NR3B1 transcriptional activity *via* β-catenin [92], reducing breast-cancer cells migration. In contrast, NR3B1-activation stimulates *Vascular-Endothelial-Growth-Factor* production and angiogenesis [93]. In ER^+^/breast-cancer cells, NR3B1 increases estrogen synthesis *via* aromatase induction [94]. As local synthesis of estrogens is fundamental for the growth of post-menopausal ER^+^/breast-cancer, NR3B1 inhibition may represent a therapeutic strategy in these patients. In breast-cancer, single study indicates that NR3B2 is a potential tumor-suppressor [95], while the potential tumor-suppressive activity of NR3B3 is supported by more studies [82, 96, 97]. NR3B3suppresses breast tumor growth and reverses the process of epithelial-to-mesenchymal transition [97].

In summary, while NR3B1 suppression/inhibition is likely to be of therapeutic value in *Her2, Basal* and post-menopausal or tamoxifen resistant *ER^+^*/tumors, activation or induction of NR3B3 may represent a viable therapeutic strategy in *ER^−^*/tumors.

### NR5A1 (SF-1:steroidogenic-factor-1) and NR5A2 (LRH-1:liver-receptor-homolog-1)

NR5A1 controls the expression of genes involved in cortisol/corticosterone biosynthesis [98], while NR5A2 play a role in hepatic bile-acids-metabolism, cholesterol-transport and glucose-homeostasis [99]. It is still unresolved whether NR5A1 is activated by endogenous ligands, as its transcriptional activity is stimulated by phosphorylation and by interactions with other proteins [98]. Nevertheless, NR5A1 and NR5A2 bind phospholipids, like phosphatidic-acid and phosphatidyl-choline. In addition, synthetic NR5A1/NR5A2 agonists/antagonists are available (Table 2). Large NR5A-mRNA amounts are expressed in adrenal-glands and testis (UNIGENE-Hs.495108), while the richest sources of NR5A2 are pancreas, adrenal-glands and liver (UNIGENE-Hs.33446).

In normal mammary-glands, NR5A2 levels are much higher than NR5A1 levels. *Basal* cancer over-expresses NR5A1 relative to the other tumor subtypes and normal tissue. In contrast NR5A2-mRNA is down-regulated in all breast-tumors, regardless of the PAM50 classification (Figure 4). The strongest NR5A2-mRNA expression is observed in *Luminal-A Luminal-B* and *Normal-like* which may be consistent with *NR5A2*-gene control by ERα [99–101].

NR5A1 has never been the object of studies in breast-cancer while data on NR5A2 are available. NR5A2 knock-down inhibits estrogens proliferative action in ER^+^/*MCF-7* cells and down-regulates ERα target-genes [99]. In ER^+^/ and ER^−^ /breast-cancer cells, NR5A2 is a mitogen and this action may involve NR5A2-dependent stimulation of *Growth-Regulation-by-Estrogen-in-Breast-cancer-1* (*GREB-1*) transcription [102]. In ER^+^/*MCF-7* and ER^−^/*MDA-MB231* cells, NR5A2 increases motility, a key process in metastatic spread [103]. Finally, NR5A2 may represent a negative prognostic marker, as a target-genes signature is associated with poor outcome in high-grade mammary-tumors [104]. Thus, NR5A2 is likely to be an oncogene and reduction of NR5A2-antagonists may produce anti-tumor effects.

## ORPHAN RECEPTORS

The fifteen members of the *Orphan-receptors* family are NRs for which endogenous-ligands are not identified. The sole exceptions are represented by NR1D1, NR1D2 and NR4A3 for which two endogenous-agonists are hypothesized.

### NR0B1(DAX-1:dosage-sensitive-sex-reversal-adrenal-hypoplasia-critical-region-on-chromosome-X-gene-1) and NR0B2 (SHP:small-heterodimeric-partner)

**Figure 5:**
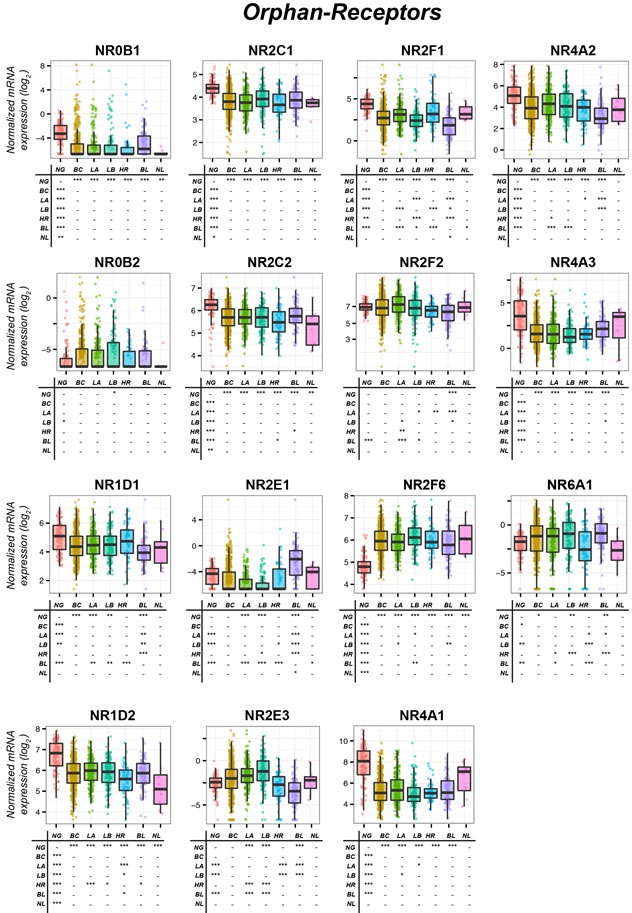
Expression of Orphan-Receptors in normal mammary glands and breast-cancer tissue The box-plots illustrate the expression of the indicated mRNAs belonging to the *Orphan-Receptors* family of nuclear receptors (NRs) in normal mammary-glands (*NG*), all breast-cancers (*BC*) and the *Luminal-A* (*LA*), *Luminal-B* (*LB*), *HER2* (*HR*), *Basal* (*BL*) and *Normal-like* (*NL*) PAM50 mammary-tumors. Underneath each box-plot, the tables show significant differences in the mRNA expression levels of each NR between the indicated groups. The results were obtained from the data available in the TCGA (The-Cancer-Genome-Atlas: http://cancergenome.nih.gov). Normalization, quantification and statistical analysis on raw sequencing counts was performed using the Limma/Voom (http://bioconductor.org) package in R statistical environment. * (adjusted *p* < 0.01), ** (adjusted *p* < 0.001), *** (adjusted *p* < 0.0001).

NR0B1 and NR0B2 are devoid of DNA-binding domains and act as co-repressor/co-activator of NR1Bs/RARs, NR2Bs/RXRs, NR1Cs, NR1Hs and NR1H4 [105, 106]. NR0B1 plays a role in adrenal-cortex development and puberty onset, while NR0B2 controls various aspects of cell metabolism. NR0B1 expression is restricted to adrenal-glands, lung and pancreas (UNIGENE-Hs.268490), while NR0B2 mRNA is measurable in liver, stomach, heart, lung and intestine (UNIGENE-Hs.427055). Mammary-glands contain low NR0B1-mRNA and NR0B2-mRNA levels and NR0B1-mRNA is down-regulated in all PAM50 breast-cancer types (Figure 5). NR0B1 is associated with ERα, PR and AR expression [107] and is a positive prognostic factor in node-negative breast-cancer, being correlated with smaller tumor-size, earlier disease-stage and increased survival [106, 107]. NR0B1 is induced by AR-activation in ER^+^/*MCF-7* breast-cancer cells and this causes aromatase down-regulation. The effect may underlay AR-ligands anti-estrogenic action. NR0B1 over-expression induces growth-inhibitory and apoptotic responses in *MCF-7* cells [108]. NR0B2 activation by CD437 exerts pro-apoptotic actions in ER^−^/*MDA-MB-468* breast-cancer cells *via* transcriptional mechanisms [109]. However, NR0B2 triggers apoptosis also *via* BCL-2 binding in mitochondria [109]. In conclusion, NR0B1 and NR0B2 are endowed with onco-suppressive properties in breast-cancer.

### NR1D1 (REV-ERBα:related-to-vERBα) and NR1D2 (REV-ERBβ:related-to-vERBβ)

NR1D1 and NR1D2 control circadian-rhythms and regulate fat deposition [110, 111]. Heme seems to be the endogenous NR1D1/NR1D2 ligand. High NR1D1-mRNA (UNIGENE-Hs.592130) and NR1D2-mRNA (UNIGENE-Hs.37288) levels are observed in many tissues. NR1D1- and NR1D2-mRNAs are down-regulated in neoplastic relative to the normal mammary tissues (Figure 5). *Basal* tumors are characterized by lower NR1D1 contents than *Luminal-A, Luminal-B* or *Her2* breast-cancers. Interestingly, the *NR1D1*-gene maps close to the *ERBB2*-gene and is often amplified in HER2^+^ tumors [112]. As for NR1D2 mRNA, the lowest levels are observed in *Normal-like* tumors. While no studies on NR1D2 in mammary-tumors are available, NR1D1 may exert onco-suppressive effects, as silencing causes reduced growth/apoptosis of HER2^+^/*BT-474* and ER^+^/*MCF-7* cells [112].

### NR2C1 (TR2:testicular-receptor-2) and NR2C2 (TR4:testicular-receptor-4)

NR2C1 and NR2C2 can form heterodimers and act as transcriptional activators/repressors of other NRs. NR2C1 and NR2C2 are involved in early embryonic development and embryonic stem cells [113]. NR2C1-mRNA (UNIGENE-Hs.108301) and NR2C2-mRNA (UNIGENE-Hs.555973) are expressed ubiquitously and are measurable in mammary-glands. NR2C1 and NR2C2 levels are lower in breast-cancer than normal tissue (Figure 5). NR2C1 is over-expressed in *Basal* relative to *Her2* and *Normal-like* tumors. In ER^+^/breast-cancer cells, NR2C1 suppresses ERα-mediated transcriptional activity, blocking ERα-binding to DNA *via* formation of an ERα-NR2C1 heterodimer. This inhibits estrogen-induced cell-growth and G(1)/S transition [114]. NR2C1 may indirectly contribute to ATRA anti-estrogenic activity in ER^+^/breast-cancer, as the retinoid controls the activity of this NR in other cellular contexts [115, 116]. NR2C1 suppresses androgen-mediated AR transactivation, which may be of therapeutic interest in AR^+^/mammary-tumors [117]. The few available data do not provide clues as to the relevance of NR2C1 and NR2C2 in breast-cancer, but it can be suggested that the two receptors are anti-oncogenic.

### NR2E1 (TLX:drosophila-tailless-homolog) and NR2E3 (PNR:photo-specific-nuclear-receptor)

NR2E1 plays a role in neuronal stem-cells homeostasis [118], while NR2E3 is involved in retinal visual function [119]. NR2E1-mRNA (UNIGENE-Hs.157688) and NR2E3-mRNA (UNIGENE-Hs.187354) expression is restricted to eye and brain and eye and muscle, respectively. Low levels of NR2E1-mRNA and NR2E3-mRNA are detectable in mammary-glands and NR2E1 is further down-regulated in breast-cancers (Figure 5). Higher NR2E1 expression levels are observed in *Basal* relative to *Luminal* tumors, while the opposite is true for NR2E3. Limited functional data on NR2E1 and NR2E3 are available and only few pertain to the breast-cancer realm. NR2E1 represses target-gene expression in neuronal-stem-cells [118], is an oncogene in glioblastoma and inhibits senescence in different cell types [120]. A recent and seminal paper by Lin *et al.* [121] supports the idea that NR2E1 is a potential drug target for the treatment of ER^−^ breast cancer. In fact high levels of NR2E1 expression in ER^−^ are a negative prognostic factor in this type of cancer. Consistent with this targeted knock-down of NR2E1 inhibits the growth of different ER^−^ breast cancer cell lines. By converse, over-expression of the nuclear receptor stimulates the formation of mammospheres, the growth and the invasive behavior of ER^−^ *MDA-MB231* cells [121]. NR2E3 is an ERα transcriptional activator [122] and it may contribute to hormone-dependent growth of ER^+^/tumors, although high NR2E3 levels are associated with favorable responses to tamoxifen. NR2E3 oncogenic action may extend to ER^−^/tumors, inducing *MDA-MB-231* cell migration *in-vitro* and metastatic spread *in-vivo* [122].

### NR2F1 (COUP-TFα:COUP-transcription-factor-1α), NR2F2 (COUP-TFβ:COUP-transcription-factor-1β) and NR2F6 (COUP-TFγ:COUP-transcription-factor-1γ)

NR2F1, NR2F2 and NR2F6 control embryonic development. Two NR2F2-protein variants are devoid of a DNA-binding domain (Table 3). The *bindingDB* database (http://www.bindingdb.org) contain several potential NR2F2-ligands, such as pyridaben. NR2F1-mRNA (UNIGENE- Hs.519445), NR2F2-mRNA (UNIGENE- Hs.347991) and NR2F6-mRNA (UNIGENE- Hs.519445) are expressed ubiquitously. Relative to the normal gland, mammary-tumors contain smaller and equal amounts of NR2F1 and NR2F2, respectively. *Basal-like* tumors express the lowest levels of both NR2F1 and NR2F2. Relative to the normal gland, NR2F6 is over-expressed in all PAM50-classified breast-cancer sub-types (Figure 5).

NR2F1 enhances ERα transcriptional activity increasing ERK-2 dependent phosphorylation [123]. NR2F1 silencing stimulates ER^+^/MCF-7 cell-growth *in-vivo* [124]. NR2F1 over-expression in *MCF-7* cells down-regulates CXCL12 and up-regulates the corresponding receptor, CXCR4, *via* activation of the EGF-pathway. This increases *MCF-7* cell-growth and cell-motility in response to CXCL12, suggesting a role for the NR2F1 in metastatic spread [125]. In breast-tumors, high NR2F2 mRNA levels are associated with better overall-survival and increased time-to-metastasis. NR2F2 silencing does not affect the growth/survival, while it increases *MCF-7* and ER^−^/*MDA-MB-231* cell-migration. NR2F2 inhibits TGFβ-dependent EMT in both cell-lines [126]. NR2F2 down-regulation is associated with anti-estrogen resistance and NR2F2 over-expression reinstates sensitivity [127]. NR2F2 over-expression in ER^−^/*MDA-MB435* cells causes growth-inhibition and G2/M phase arrest. Taken together the available results indicate that NR2F1 and NR2F2 are characterized by onco-suppressive properties in breast cancer, suggesting that strategies aimed at increasing their expression levels or aimed at stimulating their transcriptional activity are likely to be of therapeutic value. There are no studies on the significance of NR2F6 in mammary-tumors. However, given over-expression in all breast-cancer sub-types, NR2F6 may be a pro-oncogene.

### NR4A1 (NUR77:nuclear-receptor-related-77), NR4A2 (NURR1:nuclear-receptor-related-1) and NR4A3 (NOR-1:nuclear-orphan-receptor-1)

NR4A1, NR4A2 and NR4A3 may control target-gene expression in a ligand-independent manner, acting as homodimers or RXR-heterodimers. NR4As are implicated in cell-cycle regulation, apoptosis, inflammation and metabolism [128]. The only known NR4A ligands/activators are prostaglandin-A2 and 6-mercaptopurine which target NR4A3 (Table 3). NR4A1-mRNA is expressed in many tissues, including mammary-glands, although peripheral-nerves and adipose-tissue the highest levels (UNIGENE-Hs.524430). NR4A2-mRNA expression is restricted to bone-marrow, adrenal-glands, oviduct and sympathetic-ganglions,. The richest NR4A3 sources are adipose tissue, adrenal-glands and peripheral nerves (UNIGENE-Hs.279522). Mammary-glands contain similar levels of NR4A2 and NR4A3 transcripts.

Smaller NR4A1-mRNA amounts are observed in all PAM50 mammary-tumor types relative to the normal gland (Figure 5). *Luminal-A* and *Normal-like* show higher NR4A1 mRNA levels than *Luminal-B* tumors. NR4A1-agonists induce apoptosis in mammary-tumor cells [129], although NR4A1 apoptotic activity is not necessarily related to NR4A1 transcriptional activity. In ER^+^/*MCF-7* cells, the apoptotic action of a natural coumarin and Plexin-D1 [130] requires *JNK*-dependent phosphorylation of NR4A1 and translocation from the nucleus to the cytoplasm, where the NR binds and inhibits BCL-2. NR4A1 activation reduces breast-cancer cell-migration, [131], although *NR4A1-*silencing inhibits TGF-β-induced EMT suggesting an opposite effect. The effect on EMT is consistent with the observation that inflammatory cytokines induce NR4A1 and enhance TGF-β-dependent breast-cancer cell-invasiveness *in-vitro* and *in-vivo*. Thus, NR4A1 induction/activation may reduce breast-cancer growth, although this beneficial effect may be counterbalanced by increased metastatic-spread.

NR4A2-mRNA is down-regulated in all PAM50 sub-types relative to the normal mammary tissue and, consistent with data obtained at the protein level [132], *Basal* tumors contain the smallest NR4A2 amounts (Figure 5). In primary breast-cancer, NR4A2 expression is inversely correlated with lymph-node metastases and directly correlated with increased relapse-free survival, suggesting onco-suppressive properties. In spite of this, NR4A2 silencing in *Basal* cell-lines decreases xenograft growth [133]. NR4A2 inhibits aromatase expression in mammary-gland stromal adipocytes [134] and this action may have implications for breast-cancer prevention, as obesity and estrogen production are breast-cancer risk-factors. The few data available in mammary-tumors support therapeutic strategies based on NR4A2-agonists. As the NR4A2 E-region is occupied by hydrophobic molecules, which prevents synthetic ligand accessibility [135], the observation that 6-mercaptopurine activates NR4As by targeting the N-terminal portion discloses new avenues in the design of agonists [136].

Although NR4A3-mRNA is generally down-regulated in breast-cancer relative to the normal gland, *Basal* contain larger amounts than *Luminal-A* or *Luminal-B* tumors (Figure 5), confirming the results of a study showing NR4A3 up-regulation in *TN* relative to *Luminal* breast-tumors [137]. This suggests an onco-suppressive role of NR4A3 in mammary-tissue, consistent with the NR4A3 growth-inhibitory action in other cellular contexts [138, 139]. Onco-suppression in breast-cancer is supported by NR4A3 up-regulation during apoptosis in *MCF-7* cells [140]. NR4A3 induction in *MCF-7* cells by the cyto-differentiating agent, ATRA, is also consistent with NR4A3 onco-suppressive potential [141].

### NR6A1 (GCNF:germ-cell-nuclear-factor)

NR6A1 plays a crucial role in embryonic-stem-cell (ESC) homeostasis [142]. In ESC, NR6A1 is a positive determinant of pluripotency being down-regulated by the differentiating-agent, ATRA [99]. In adults, NR6A1-mRNA tissue-specific expression is limited to ovary and testis (UNIGENE-Hs.586460). Mammary-glands contain very low amounts of NR6A1 which are generally up-regulated in breast-cancer (Figure 5). The PAM50 subtypes showing the highest levels of NR6A1 up-regulation are *Basal* and *Luminal-B* tumors. The first observation is supported by a recent study indicating NR6A1 gene-expression enrichment in TN/ and ER^+^/tumors [143]. These data suggest that NR6A1 may be endowed with oncogenic properties in breast-cancer.

## CONCLUSION

Integration of the expression and functional data available allows a reliable prediction of the onco-suppressive or oncogenic role played by many of the NRs considered in breast-cancer (Tables 1, 2, 3). Within the *Lipid-Sensors* group, NR1C3, NR1H2 and NR1H3 are likely to play an onco-suppressive action. NR1F1, NR2A1 and NR3B3 (*Enigmatic-Orphans*) as well as NR0B1, NR0B2, NR1D1, NR2F1, NR2F2 and NR4A3 (*Orphan-Receptors*) seem to exert a similar activity. These NRs represent viable candidates for the development of therapeutic strategies aimed at increasing their expression or activating them in tumor cells. The availability of pharmacological agonists for NR1C3, NR1H2, NR1H3, NR1F1, NR0B2, and NR4A3 should boost pre-clinical studies in this direction. For all the remaining NRs, efforts should be oriented towards the design and synthesis of selective and high-affinity agonists. Except for NR1D1 and NR2F1, whose levels are significantly lower in *Basal-like* relative to the other PAM50 subgroups, the expression profiles of the transcripts encoding the above mentioned NRs do not indicate any expression specificity in terms of breast-cancer subtypes. Nevertheless, as indicated in Table 2 and on the basis of the available literature, NR1F1 may be of particular significance as a therapeutic target in ER^−^ breast cancer. The group of NRs endowed with potential oncogenic properties in breast-cancer is smaller and consists of the *Lipid-Sensors*, NR1C2 and NR1I2, the *Enigmatic-Orphans*, NR1F3, NR3B1 and NR5A2, as well as the *Orphan-Receptors*, NR2E1, NR2E3 and NR6A1. To obtain anti-tumor effects, oncogenic NRs should be targeted with selective antagonists, reverse-agonists or agents/strategies capable of reducing their expression in breast-cancer cells. At present only synthetic antagonists targeting NR1C2, NR1I2 and NR1F3 are available. On the basis of the expression profiles in the PAM50 subgroups, we propose that NR1C2, NR1I2 and NR2E1 are pharmacological targets of particular interest in *Basal-like* tumors. In the case of NR2E1, this is line with the evidence present in the literature which indicates that targeting of the receptor is particularly promising in ER^−^ mammary tumors [121]. A similar relevance in ER^−^ is predicted also for NR1F3, as indicated in Table 2. Similar considerations suggest that NR1C2 and NR3B1 may be a useful targets in *Her2* breast cancer as well (Tables 1, 2). Finally the interest of NR3B1 may not be limited to this last group, as it may extend to ER^−^ mammary tumors regardless of HER2-positivity (Table 2).

In the case of both onco-suppressive and oncogenic NRs there are some general points that should be considered and were touched upon in this review. Studies focusing on NR-targeting should be aimed at establishing the therapeutic potential in specific types of breast cancer given the heterogeneity of the disease. It is, indeed, highly unlikely that each of the identified NRs plays the same role in all mammary tumor-subtypes and targeting it results in similar anti-tumor effects, as recently demonstrated for the activation of RARs by ATRA and derived retinoids [5]. Thus, studies should not be limited to evaluating differential effects in the ER^+^/ and ER^−^/cellular context, as traditionally done, but should take into consideration other identified breast cancer sub-groups such as the PAM50 classes used in this review. In addition, when different protein-variants of a specific NR are known, it is important to gather information as to the specific forms predominantly expressed in each breast-cancer subtype. In fact, this type of information is not available and should be gathered, as different protein-variants may have opposite effects in terms of oncogenic or onco-suppressive activity. Finally, the side effects potentially triggered by targeting a specific NR in breast-cancer should be considered before designing any targeted therapeutic strategy. With respect to this, a preliminary analysis of the data available on the tissue-distribution and physiological function of each NR is likely to be helpful in the selection of the specific NR to be targeted.
